# Marshall Islands Pinworm

**DOI:** 10.7759/cureus.4722

**Published:** 2019-05-22

**Authors:** Sindhura Kolli, Sree S Kolli, Mel A Ona

**Affiliations:** 1 Internal Medicine, The Brooklyn Hospital Center, New York, USA; 2 Medicine, University of Vermont, Burlington, USA; 3 Gastroenterology, Pali Momi Medical Center, Honolulu, USA

**Keywords:** pinworm, colonoscopy, enterobius

## Abstract

Pinworm infections are usually under the spectrum of the Infectious Diseases department, however, they can fall into a gastroenterologist’s lap when found incidentally during a screening colonoscopy. This case expands on the epidemiology, clinical presentation, diagnosis, and treatment of pinworms in the patient and household.

## Introduction

During screening colonoscopy, a number of pathologies are expected: polyps, ulcers, bleeding, hemorrhoids, and more. Rarely, is a pinworm encountered during a routine screening in an asymptomatic patient. A patient from Marshall Islands, however, surprised the endoscopist both with the presence of a pinworm during a colonoscopy and their asymptomatic presentation. This case demonstrates an incidental pinworm finding and delves into its clinical presentation, diagnosis, and treatment.

## Case presentation

**Figure 1:**
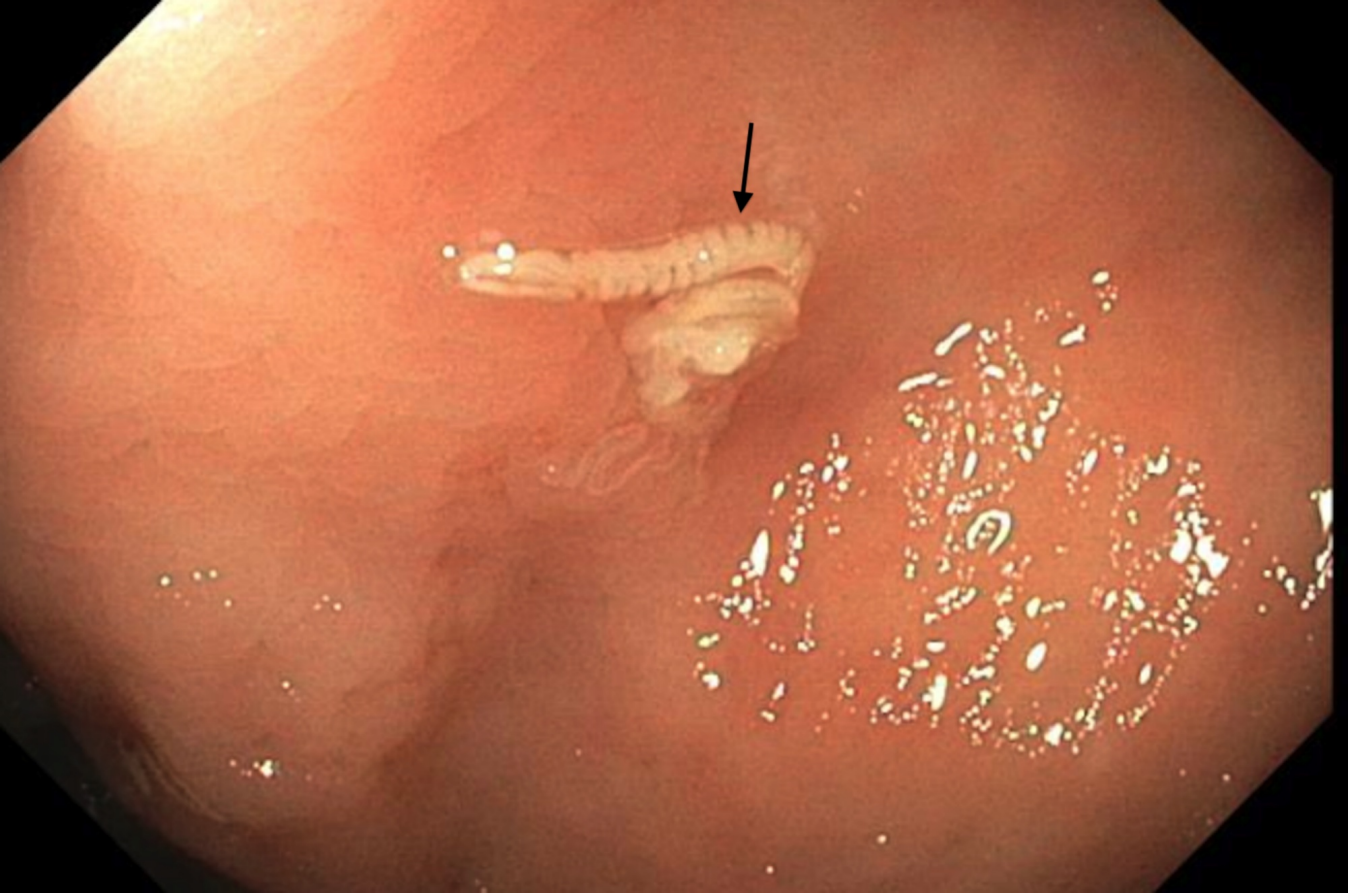
Pinworm discovered incidentally during screening colonoscopy.

**Figure 2:**
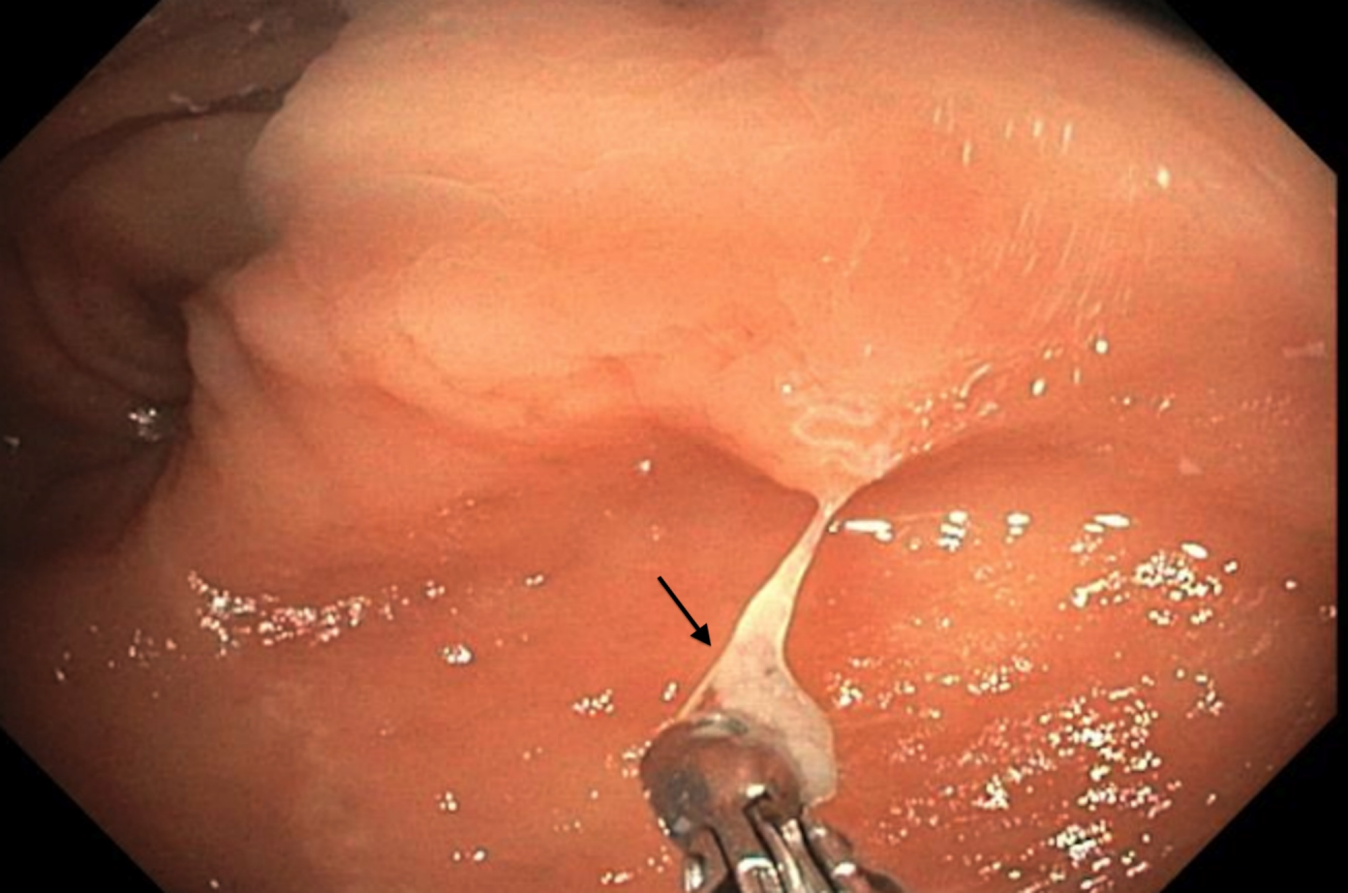
Pinworm retrieved with cold biopsy forceps.

A 64-year-old female patient from the Marshall Islands presented for a routine average-risk screening colonoscopy for colorectal cancer. She had no presenting symptoms or complaints and had no family history for gastrointestinal malignancy. Vital signs and labs were within normal limits. Colonoscopy demonstrated adequate bowel preparation with Boston Bowel Preparation Score of nine which made for clear visualization of a whitish-colored worm within the cecum (Figure 1). It was retrieved with the cold biopsy forceps and sent for pathology (Figure 2).

Pathology revealed a parasitic organism consistent with pinworm also known as *Enterobius vermicularis*. Patient was treated with over-the-counter pyrantel pamoate (11 mg/kg) two doses given two weeks apart. Patient was asymptomatic to begin with and posed no more symptoms or complaints following treatment.

## Discussion

The prevalence of intestinal parasites is high with estimates of over one billion people affected [1]. The highest prevalence is found in African, Asian, and Latin American countries [2]. Populations most vulnerable to these infections have risk factors such as poor sanitation practices and overcrowding often found in developing countries [1]. People affected in the United States are commonly immigrants from developing countries or have a compromised immune system [3].

*Enterobius vermicularis* or pinworm is the most common helminth infection in the United States [4]. Pinworms are easily transmissible between those living in close quarters, sharing clothing or sleeping spaces [1]. Pinworm eggs are ingested and proceed to hatch in the small intestine [1]. The larvae migrate to the large intestine and are found primarily in the cecum and appendix [1]. Adult females mature and travel to the perianal region at night laying eggs and infecting clothes, sheets, and fingernails following intense pruritus in perianal region [1]. About 30% of infected patients exhibit symptoms with clinical presentations include pruritus ani and appendicitis [5-6]. Our patient was asymptomatic possibly because the larvae had not yet migrated to the perineal region and was not situated near the appendix. Diagnosis in symptomatic patients is usually done by placing a piece of Scotch tape in the perianal region at night or early morning and then placed on a glass slide to visualize eggs under a microscope or microscopy of samples taken from under fingernails. Stool samples are not recommended as eggs and worms are generally minimal in stool and serology tests are not available for pinworm [1]. The diagnosis in our asymptomatic patient was made incidentally when the pinworm was visualized during a screening colonoscopy. Treatment of pinworm is with over-the-counter pyrantel pamoate or with a prescription of mebendazole or albendazole. Usually two doses are administered two weeks apart in order to broaden coverage for newly hatched eggs within the two week span. Treatment is recommended for the close contacts in households and work institutions as well [7].

## Conclusions

During a routine colonoscopy, anything should be expected and appropriately dealt with it. In this case, an incidental pinworm in a completely asymptomatic patient was demonstrated, which initiated prompt treatment with a cheap over-the-counter medication to both the patient and her household preventing a transmissible disease from spreading. It reminds every endoscopist to be prepared to find anything during a routine colonoscopy.
